# Vehicle Detection and Recognition Approach in Multi-Scale Traffic Monitoring System via Graph-Based Data Optimization

**DOI:** 10.3390/s23031731

**Published:** 2023-02-03

**Authors:** Grzegorz Wieczorek, Sheikh Badar ud din Tahir, Israr Akhter, Jaroslaw Kurek

**Affiliations:** 1 Department of Artificial Intelligence, Warsaw University of Life Sciences, 02-787 Warsaw, Poland; 2 Department of Software Engineering, Capital University of Science and Technology (CUST), Islamabad 44000, Pakistan; 3 Department of Computer Science, Bahria University, Islamabad 44000, Pakistan

**Keywords:** multi-scale traffic monitoring (MSTM), artificial neural network (ANN), histogram of gradient (HoG), leave-one-subject-out (LOSO)

## Abstract

Over the past few years, significant investments in smart traffic monitoring systems have been made. The most important step in machine learning is detecting and recognizing objects relative to vehicles. Due to variations in vision and different lighting conditions, the recognition and tracking of vehicles under varying extreme conditions has become one of the most challenging tasks. To deal with this, our proposed system presents an adaptive method for robustly recognizing several existing automobiles in dense traffic settings. Additionally, this research presents a broad framework for effective on-road vehicle recognition and detection. Furthermore, the proposed system focuses on challenges typically noticed in analyzing traffic scenes captured by in-vehicle cameras, such as consistent extraction of features. First, we performed frame conversion, background subtraction, and object shape optimization as preprocessing steps. Next, two important features (energy and deep optical flow) were extracted. The incorporation of energy and dense optical flow features in distance-adaptive window areas and subsequent processing over the fused features resulted in a greater capacity for discrimination. Next, a graph-mining-based approach was applied to select optimal features. Finally, the artificial neural network was adopted for detection and classification. The experimental results show significant performance in two benchmark datasets, including the LISA and KITTI 7 databases. The LISA dataset achieved a mean recognition rate of 93.75% on the LDB1 and LDB2 databases, whereas KITTI attained 82.85% accuracy on separate training of ANN.

## 1. Introduction

Computer vision is a highly utilized study field in technologies such as industrial automation, robotics, characterization recognition and identification, human/machine interface, text analytics, and motion detection [1]. Identification of moving objects is an interesting subject of study in computer vision and image processing applications. Therefore, identifying a moving object is crucial for a wide range of applications, including video surveillance systems. As the population rises, the smart transport system adapts accordingly. Therefore, significance and application become mandatory. 

Security is a primary concern over the motorway public transport system. According to the research and published reports of many researchers in the past year, a growing number of tragic incidents have happened, and the number is rising daily. The purpose of the smart transportation program is to enhance traffic efficiency—the convenience and security of movement—which enables an automated, financial, and realistic solution. Alternative uses include congestion control, independent navigation, automated technology, and vehicle reconfiguring. Monitoring and analyzing automobiles, thereby capturing data on the driving patterns of the transfers of information and gaining information on the magnitude of the number of automobiles in a certain region, can reduce several pollutants [2]. 

Various studies experimented with tracking and locating moving cars using video monitoring. Typically, the video monitoring system incorporates both stationary and mobile objects. The primary objective is to determine the physical motion of a rotating shaft in a certain area. Objects within the video system may be recognized using moving object approaches [3,4]. This approach allows for the automatic detection and surveillance of moving traffic within a video frame. The classification of vehicular traffic is utilized to establish a relationship between objectives or object pieces in two sequences and to obtain characteristics about the target, such as its trajectory, acceleration, and orientation. Objects are discovered frame by frame in a movie by using the scanning of passing vehicles. It has multiple applications, including video surveillance, network monitoring, and people tracking [5]. On the other hand, several variables in the past several years, such as speed, consistency, and small distance, have become bothersome automobile traffic problems. The climate and heavy emphasis also significantly impact achieving correct detection findings [6].

Consequently, many locations and towns have multiple video cameras to monitor and record traffic occurrences. Intelligent traffic analysis is superior to traditional and applied mechanical time-based and sensor-based evaluation techniques [7]. The model accounts for the changing sizes of the items to be discovered. Several vision-based characteristics that might be added include the object’s structure, geometry, outline, width and height margins, darkness, lights, annular spotlights, HoG characteristics, and Haar characteristics. Attributes can also be employed for image recognition; for instance, edges, parallelism, and headlight permutations can be deployed as aggregated features for darkness prevention and detection. The issues with camcorder vision are not rectified. The lens is responsible for dealing with eyesight issues. Thus, no vision-based characteristics are presented. Efficient vehicle identification and concentration estimation play a significant role in facilitating transportation. Identification and tracking are the two main steps required here—the precise recognition of cars amid other things in the scene [8,9,10]. Additionally, the cars presented may be of varying sizes and shapes. Consequently, the initial phase of object recognition has unique challenges, which greatly encouraged the investigation.

This paper proposes an enhanced method for automobile automatic recognition and classification. Many neural algorithms use different processing approaches or incorporate a range of techniques to provide a better system, which can result in a time-consuming and costly task that might hinder real-time inspection and analysis. The technique’s purpose is to identify automobiles within the provided traffic data [11]. The collection consists of numerous forms of roadside objects, including several types of cars and other things, such as roads and passengers. Some automobiles are far away and minimal, and others are nearby and large. In this paper, we introduce an improved MSTM system that is intended to detect and recognize present vehicles in a dense traffic environment. Our MSTM-based system involves different steps: data acquisition, frame conversion, object shape optimization, extracting regions of interest, feature abstraction, graph mining optimization, and classification. First, we acquired scene data from two benchmark datasets. Then, we transformed video data into image data for preprocessing. The acquired data is further filtered via the median filter and a background subtraction strategy. We optimized the image data’s outer shape after denoising it to obtain better results. Therefore, we employ both the outer layers of change detection and the Gaussian mixture model. Next, the silhouette recognition process is performed using consecutive edge detection and ridge data combination techniques. Furthermore, we employed two sophisticated methods for feature abstraction, including energy and dense optical flow features. Finally, a graph mining strategy is adopted to select the optimal data and cater to the ANN for detection and recognition. 

Our proposed MSTM identifies the vehicle that are visible in given data frame and set of images. There are other methods with this efficient detection. However, this methodology delivers a higher rate of prediction potential with less computation power. Detecting long-distance vehicles that appear to be delicate things is an additional significant achievement. The main contributions of this paper are as follows:

- Targeted at complex datasets, preprocessing, and optimized outer body shape extraction approaches are performed.
- Robust approaches such as denoising, object shape optimization, and feature extraction are applied to extract useful information in feature extraction methods.
- To smooth the process of detection and recognition of ANN, a graph mining strategy is employed for better feature selection.
- Additionally, two benchmark datasets—LISA and KITTI 7—were subjected to an extensive examination for a multi-scale traffic monitoring system. The results of the experiments show a higher recognition rate that also exceeds sophisticated systems.

The remaining parts of this research study are as follows. The complete details of related and previous work are discussed in Section 2. Section 3 describes the proposed method comprises preprocessing, background subtraction, features extraction approach, and data mining and classification procedures. Section 4 illustrates the experimental setup, details of experiments, results, and comparison with other state-of-the-art approaches. Finally, Section 5 describes the conclusion of the proposed paper, with limitations, scope of the study, feasibility direction and provides few future recommendations. 

## 2. Related Work

Since the automobile’s appearance is warped and influenced by numerous circumstances, vehicle recognition and identification are essential but challenging operations. First, more vehicle varieties are being produced as new automobile models are frequently advertised. Then, there are also many differences between some automobiles. Lastly, many discrepancies in automotive scenes are also caused by various road conditions, weather, lighting, and equipment types. 

Currently, the majority of research that has been published mainly focuses on grouping vehicles into broad groups, including motorbikes, automobiles, minibuses, or tankers [12]. However, more versatility is needed to meet user requests. To obtain the data indicating the car’s manufacturing company, various researchers analyzed the identification and recognition of automobile logos utilizing frontal automobile photos [13]. Some scientists recently modified the extraction of features and classification models to categorize automobiles into accurate classes. Munroe employed canny margins as the selected feature for object recognition and tried various classification techniques: k-NN, neural network, and decision tree [14]. There were 30 examples in each of the 5 categories that made up the dataset. In Clady’s method [15], Sobel boundaries are extracted and focusing elements are acquired. Petrovic and Cootes described an analysis of extracted features and recognition to build a rigid structured recognition framework for the automatic classification of automotive kinds [16]. 

In order to describe a class of vehicle for cross-car class identity that is resilient to illumination variations and brightness for verification, P. Negri et al. [17] devised an aligned outline point-based voting algorithm. Wavelet transforms, rapid Fourier transforms, and discrete curvelet transforms were examined by Kazemi et al. [18] in the classification of five car models using convert picture features. Zhang et al. [19] first investigated two techniques for extracting features for video description, including the spectral analysis and the pyramid histogram of centered gradient. Zhang then put forth an accurate classification strategy for identifying different forms of transport using cascade classifier ensembles. Hannan et al. [20] introduced vehicle detection and identification using image processing for vehicle tracking. In order to obtain good classification accuracy, this method applies the fast computational model as a main classification and the traditional neural network as a final classification algorithm. 

For nocturnal vehicle recognition, taillight properties are frequently exploited. At dark, the taillights are predominantly red, making it simple to recognize them from the surroundings. The distribution of taillights shows the ROI of automobiles, and it is simple to identify the placement of the headlights from the red channel of the image-by-image processing [21]. Qian et al. [22] integrated the SIFT characterization with the classification model for cross-identification and tracking. The suggested mixture enables effective navigation in challenging circumstances. However, employing these properties in practical application domains, such as transportation camera surveillance, is constrained by the breadth of the reference image, its computational complexity, and its poor response to different lighting conditions. Shujuan et al. [23] combined a classification method and the Moderate AdaBoost classification with a blend of pseudo-Haar and SURF properties for real-time object recognition. The SURF descriptor lacks much usefulness for detecting motor vehicles considering its resilience and exact verification process due to its unpredictability despite changes in light. 

Elkerdawi et al. [24] utilized pseudo-Haar features as the driving force behind a cascade classification utilizing the AdaBoost algorithm to find and follow vehicles in a roadway camera situation. Miller et al. [25] addressed the use of pseudo-Haar features for detection and tracking and a hidden Markov model to characterize the speed of the vehicles. Additional boosted-HOG characteristics have been put forth by Sun and Watada [26] for detecting cars and humans in still photos of transportation. The significantly increased features are derived after training the AdaBoost algorithm on data from the learning foundation. Subsequently, using its boosted-HOG properties, a quadratic SVM is trained. The latter combines the benefits of the HOG classification and the AdaBoost algorithm. Finally, dominant structures of the histograms of oriented gradients (DPHOG) are applied in detection and tracking by Natthariya et al. [27]. For the protection of motorists and passengers in intelligent transport detection techniques, DPHOG has two factors that impact computational speed and precision during the classification process of detection and tracking. The DPHOG uses ideal dimensions of the automobile and non-vehicle photographs to select the dominating sequences from HOG characteristics. Girshick et al. [28] suggested computing a fixed CNN primitive matrix for each instance proposition section to find and partition artifacts. Sermant et al. [29] evaluated the R-CNN in their proposed system. In addition, the researchers observed that on a Pascal VOC basis, R-CNNs beat HOG features in image categorization. Two explanations were provided by Girshick et al. [28] for this recognition rate. The initial observation was that all categories and utilize the same CNN sceneries. Moreover, compared to other methods, such as encoding a spatial pyramidal bag, the matrix of CNN troglodytes is smaller.

Yu et al. [30] suggested a length-based technique for classifying moving traffic in multi-lane transportation image sequences in actual environments. The longitudinal translation is supplemented by feature extraction, edge-based shadowing eradication, and binarization feature extraction methods to categorize vehicles. Meher et al. [31] developed a technique to enhance the performance of vision-based VC by detecting and eliminating shifting disturbances. The method’s possibilities and efficacy were compared to those of existing techniques. Occlusion management is an image-processing operation that tracks an automobile when it is partially covered. Using obstruction processing tracking and one-class SVM (OC-SVM) segmentation, Moutakki et al. [32] demonstrated a technique based on opacity handle recording. Pupo et al. [33] constructed an existing security system to detect and count automobiles using the SVM classification paradigm and opacity control. Oriented gradient statistics followed by an SVM are used to categorize automobiles according to their category. The main significance of this paper and its emphasis are on preprocessing and optimal outer body contour extraction procedures, which are performed on complicated datasets. In feature selection methods, robust methods are employed to gather meaningful information. Node extraction and artificial neural networks implement data processing and optimization. In addition, two baseline datasets, LISA and KITTI 7, were submitted for a thorough analysis for the multi-scale traffic tracking system. Tests show a greater classification performance that surpasses even advanced systems.

## 3. Material and Methods

**Figure 1:**
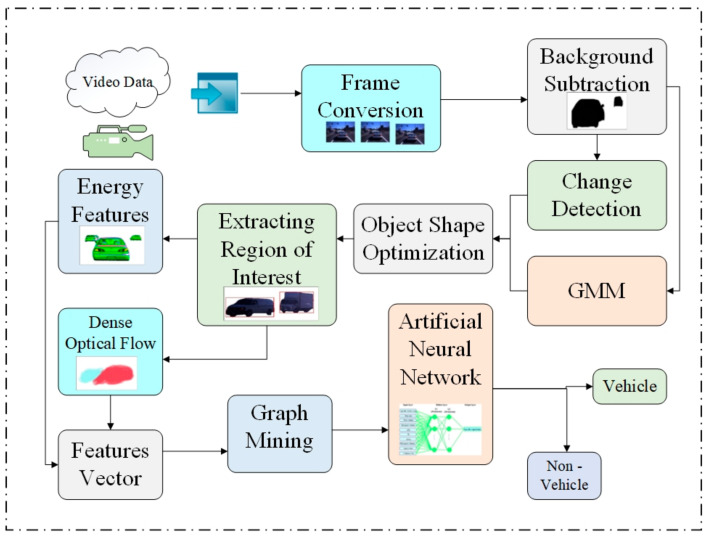
The suggested vehicle detection framework process diagram.

We initially converted the video data to images. Then, we lowered the size of the converted images, reduced image deformation, and enhanced image clarity. The subsequent phase involved detecting the region of interest from many configurations and extracting energy and dense optical flow patterns. Following this, we had to tweak the data for more efficient measurement. To accomplish this, we used graph mining. Subsequently, we classified using an artificial neural network. Figure 1 provides a graphical representation of our entire approach.

### 3.1. Frame Conversion

**Figure 2:**
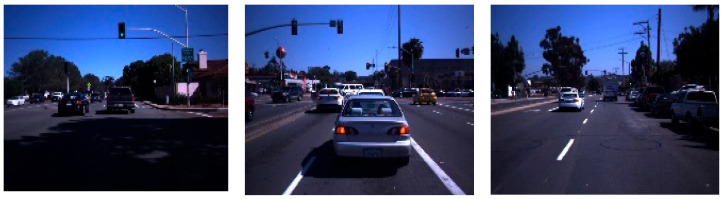
Example results of pre-processing.

Before implementing vehicle identification and recognition, we utilized a number of sequential and cost-saving preprocessing approaches. This involves the first transformation of video sequences into picture data. These photos are always 470 by 360 pixels in size. Figure 2 depicts the outcomes of converting video data.

### 3.2. Background Subtraction

**Figure 3:**
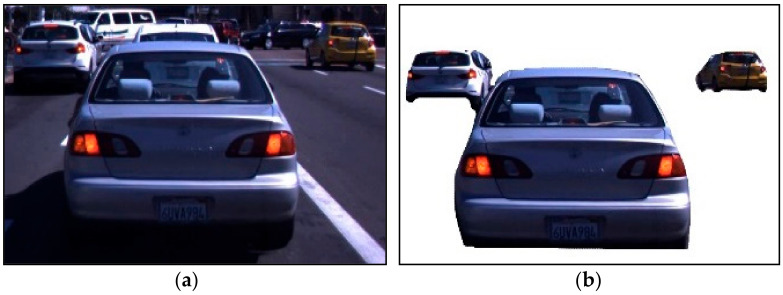
Example results of preprocessing: (**a**) denoised image and (**b**) background-subtracted image.

The next step is noise reduction and subtracting the irrelevant information in terms of background. The images are then denoised via the median filtration process. Median filtering is performed to recognize misshapen pixels in images and substitute them with the median index. We used a 6 × 6 grid to reduce noise. The mathematical illustration of the median filter is formulated in Equations (1)–(3):

(1) $$Medf\;\left(I\right)=Medf\{I_{m})\;$$

(2) $$X1=\frac{I_{m}\left(n+1\right)}{2};n\;is\;odd$$

(3) $$X2=\frac{1}{2}\left[I_{m}\left(\frac{n}{2}\right)+I_{m}\left(\frac{n}{2}\right)+1\right]$$

where *I_1_*, *I_2_*, *I_3_*,…, *I_n_* is the command of the head-to-head pixels. All existing pixels of the given pictures must be prepared in order. Afterward, the classification of the pixels and the procedure of the selected pixels is $I_{m1}<I_{m2}<I_{m3}<I_{mn}$ where n is generally abnormal. Figure 3 shows the results of noise reduction, data preprocessing and background subtraction. 

### 3.3. Object Shape Optimization

**Figure 4:**
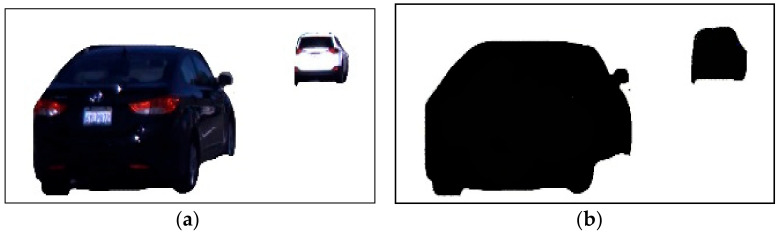
Example results of object shape optimization: (**a**) background-subtracted image and (**b**) optimized shape in binary format.

In this subsection, we optimized the vehicle’s extracted outer shape to get more accurate results. We manage both outer layers from change detection and the Gaussian mixture model. Then, we apply the addition process for both input images and store them in the next frame. Figure 4 shows the results of the optimized vehicle silhouette in RGB and binary image format. 

### 3.4. Extracting Region of Interest

**Figure 5:**
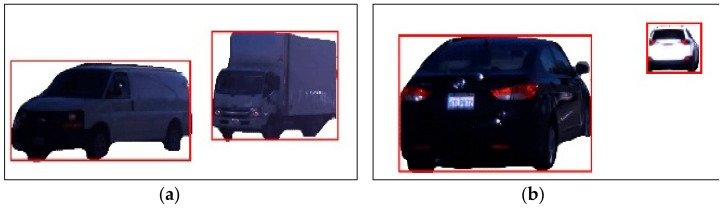
Example results of extracting region of interest. (**a**) front (**b**) back.

The extraction of the region of interest and vehicle’s silhouette identification process entails two phases [26]: consecutive edge detection and ridge data combination. In the binary edge departure procedure, second limitations are improved from the RGB outlines shaped in the aforementioned preprocessing phase. Applying space conversion, the contiguity maps are produced on the borders (see Figure 5), while in the ridgeline statistics formation step, the native optimum is assimilated from the pre-computed charting to produce ridge statistics along the dualistic apexes. The mathematical description of vehicle’s detection is

(4) $$\gamma K=n\;\sum \;x=1\;\left|\left|\alpha x\right|-\left|\beta \right|\right|\;where\;K=1,\;2,\;3,\;4\;$$

where α represents the centroid argument of the paths stored in the confusion table, *β* means the new arcs of the test statistics, and *γ* denotes the objectivity amongst the stored values of the confusion table and the original trajectories.

### 3.5. Feature Extraction

In this step, we provide the details of feature extraction approaches for vehicle recognition over state-of-the-art datasets. We applied two comprehensive approaches for extraction of features: energy and dense optical flow features. Algorithm 1 defines the entire methodology for extracting the features.
**Algorithm 1.** Features Extraction.Input: Input_data  Output: Feature_vect $\left(fe_{1,}fe_{2,}\ldots \ldots .fe_{n}\right)$
   *Extracted_features_Vector* ← []
 Data← GetData_F_F()  Data_size_F1 ← GetData_F1_size()  Procedure PAP(Video, Images) 
Features_Vectset← []  Denoise_Input_Data ← Pre_processing()  Sampled_Data(DenoiseData)  While exit invalid state do 
$\;\;\left[Ef,\;Dof\right]\leftarrow$ ExtractlFeatures(sample data) 
Feature_Vect← [] $\;\;\;\;\;\;\;\;\;\;\left[Ef,\;Dof\right]$
 Return MainfeaturesVector

#### 3.5.1. Energy Features

**Figure 6:**
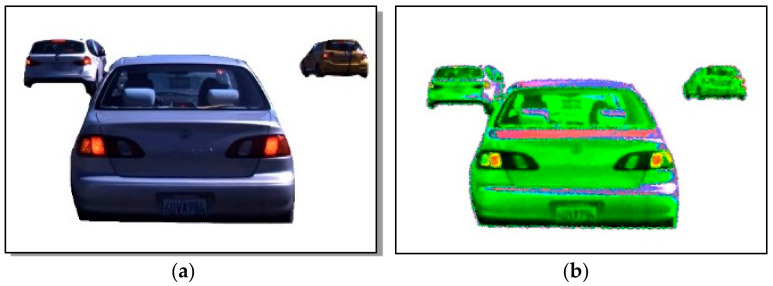
Example results of energy features: (**a**) background-subtracted image and (**b**) energy features.

The context-aware energy characteristic E(t) analyzes the power index-based matrix using a set of [0–10,000] indices across a recognized silhouette (see Figure 6). The retrieved matrix is subjected to a predefined threshold, which converts the result to an ID vector. Equation (5) illustrates the relationship between the energy feature vector and is written as follows:

(5) $$E\left(t\right)=\sum_{0}^{m}lv\left(m\right)\;$$

where $E\left(t\right)$ is energy vector, M is an index number, and In Vis RGB is the values of the index pixel.

#### 3.5.2. Dense Optical Flow

**Figure 7:**
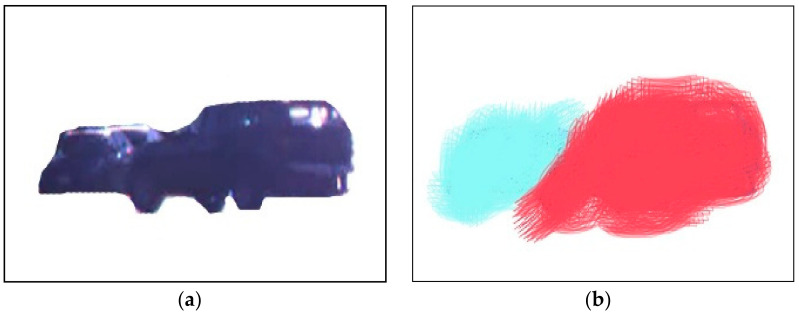
Example results of dense optical flow features: (**a**) background-subtracted image and (**b**) example results of dense optical flow.

Discretization methods that decrease nourishment things of the category are used dense optical flow. We applied this entirely in the datasets and discovered the optical flow values. Dense optical flow delivers the color diagramming on all the compressed flow regions, which is expressed as:

(6) $$Q_{1}\left(K_{1}\right)=Q_{1}^{M}(K_{1})+\partial Q_{1}^{U}(K_{1}),$$

where ∂ is a weight limitation and $Q_{1}^{M}(K_{1})$ and $Q_{1}^{U}(K_{1})$ shows the corresponding functions. The corresponding formulation is

(7) $$Q_{1}^{M}(K_{1})=\int_{\omega_{1}}(a_{1}(p_{1})-a_{2}\left(h_{1}(p_{1}\right)){)}^{2}dp_{1}$$

where $Q_{1}^{M}(K_{1})$ shows the expectations of dense structures and Figure 7 demonstrates the dense feature’s consequences.

### 3.6. Data Optimization: Graph Mining

As features are extracted from the complete dataset, the procedure performs the input features vector reduction and finds the related values for data optimization, which reduces operational expenses and improves precision. To include related input values that are also subjected to statistical foundations and indications, academics can achieve a high retrieve prediction performance by employing the graph mining process. [34]. Combining methods and techniques for data acquisition, predicting database systems, and generating an orderly and convincing graph for clustering and classification, graph mining is a technique for recognizing patterns. Algorithm 2 describes the complete operation of graph mining.
**Algorithm 2.** Data Mining via the Graph Mining Approach.Input: All Features (Af)  Output: Mined_data $\left(Mdt\right)$
*All_feature* ← []  for i = 1: k do  Read_Data: Q→(Af)  Tree_Creating: TC_tree(Q→0)  Read_Data: to find min R(min) and max R(Max)  Find_next_node: R(Af→ next_node)  Find Mutual_node: apprise_the_list  Mine_the_date: min(Tree,apprise)  Restrictive_TC_tree:Produce_the_tree(mining)  end  return Optimized Data {OD}

### 3.7. Vehical Detection: Artificial Neural Network

This section discusses the ANN approach to classification. An ANN is a collection of multiple perceptrons or neurons on each stratification; when necessary data is categorized in the forward broadcaster, this is known as a feed-forward neural network [35]. The processing elements, the hidden layers, and the output variable constitute the core structure of an ANN. The input layer accepts raw data, the concealing levels execute arithmetic on the entering data, and the artificial neuron obtains outcomes. In machine learning algorithms, each layer is responsible for learning the mathematical weights that are produced at the culmination of the learning process. The ANN approach is good for image data, text descriptions, and probability tables challenges. The advantage of ANN is its capacity to cope with transfer functions and to learn characteristics that map any input to any output for any data. The links between neurons train their neural network with exponential properties, allowing the network to learn any complex relationship between output and input data. 

**Figure 8:**
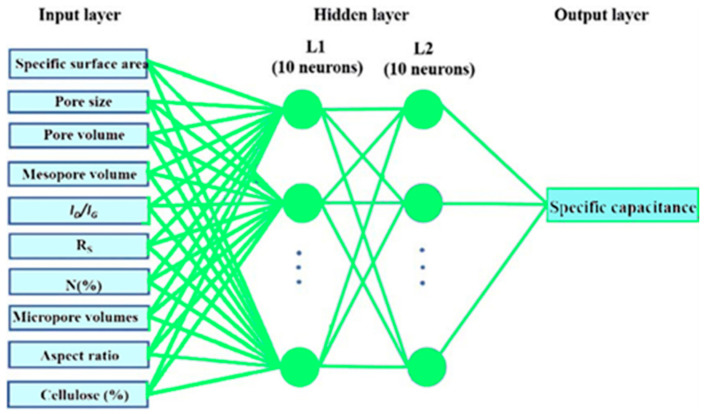
The model overview and diagram of artificial neural network.

Numerous researchers employ ANNs to analyze complex relationships, including the coexistence of mobile and WiFi connectivity in licensed spectra and then transfer the optimized features vector to ANN for classifications and segmentation; Figure 8 illustrates the system of ANN.

## 4. Experimental Evaluation and Settings

The leave-one-subject-out (LOSO) cross-validation method was incorporated to test the performance of the proposed MTMS method via two publicly accessible benchmark datasets, namely the LISA and KITTI databases.

### 4.1. Dataset Description

**Figure 9:**
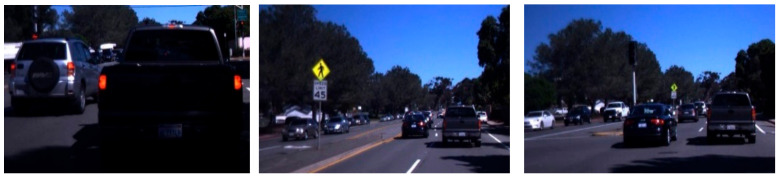
The example images of LISA dataset.

The LISA dataset [36] involves three-color consecutive frames shot at various times of day and lighting conditions: daylight, afternoon, bright, and hazy. Different traffic scenarios, including highway and urban, with variable driving conditions ranging from mild to heavy. Figure 9 shows the example images for the LISA dataset.

**Figure 10:**
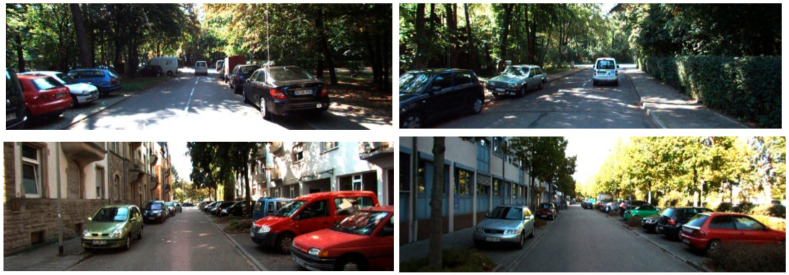
The example images of KTTI dataset.

KITTI is among the most often used datasets in robotic manipulators and automated vehicles [37]. It contains hours of videotaped traffic events captured with several screen protectors, such as increased RGB, grayscale photogrammetry, and 3D infrared sensors. However, despite its prominence, the resource does not provide feature extraction contextual information. Meanwhile, a number of researchers have annotated portions of the database to meet their needs. Figure 10 shows the example images for KITTI dataset.

### 4.2. Hardware/Software Environment

MATLAB (2021a) and Google Colab were utilized for all developments and computations. The computing machine was fused with Intel (R) Core i5- 10210U CPU GHz running 64-bit Windows 11 Pro. The notebook featured with a 16 GB of RAM and 1.6 GHz processor. LOSO was used to analyze the accuracy of proposed approach.

### 4.3. Results and Comparison

The RGB sequences recorded from the front image camera in the LISA dataset involve three databases. First, LDB1, comprising 1600 successive image frames, was recorded on a highway during a bright evening rush hour. Numerous automobiles on the road were impacted by varied lighting. Second, LDB2, comprised of 300 consecutive frames, was captured on a cloudy metropolitan road in the early morning hours. That is, the set has inadequate lighting. Third, LDB3, comprised of 300 image sequences, was recorded on a road on a sunny and clear afternoon. However, we tested our proposed system on LDB1 and LDB2 databases.

To analyze the robustness of our proposed approach, we evaluated our system with other state-of-the-art methods defined in [38]. The performance indicators comprise the true positive rate (TPR), false detection rate (FDR), and other evaluation metrics.

(8) $$\mathrm{True}\mathrm{Positive}\mathrm{Rate}\;(\mathrm{TRP})=\frac{Recognized\;Vehicles}{Total\;no.\;of\;vehicles}$$

(9) $$\mathrm{False}\mathrm{Positive}\mathrm{Rate}\;(\mathrm{FRP})=\frac{False\;detection\;of\;Vehicles}{Total\;no.\;of\;sequence\;processed}$$

(10) $$\mathrm{Precision}=\frac{TPR}{TPR+FPR}\;$$

(11) $$\mathrm{Recall}=\frac{TPR}{TPR+FNR}$$

(12) $$F-measure=\frac{TP+TN\;}{TP+FN+TN+FP}$$

As demonstrated in Table 1 and Table 2, our system’s TPR reached 95.62% on LDB1 database and 91.89 % on LDB2 database 2. In addition, our false positive rate, which is 4.2% on LDB1 and 13.14% on dataset 2, is significantly lower than that of sophisticated techniques. More specifically, our proposed system attained better performance on the given five performance metrics.

We split our studies into two components and selected the KITTI dataset for our experiments. Initially, we carried out experiments on the seven categories including *pedestrain, cyclist, car, van, tram, truck, and misc*. As truck, van, and car belong to car class and have many similar characteristics, we combined truck, car, and van into an individual class and conducted experiments on these five classes.

In next step, we individually trained the ANN classifier optimized by graph mining on our benchmark dataset. Table 3 represents the evaluation metrics (precision, recall, and F-measure) of the KITTI dataset.

**Figure 11:**
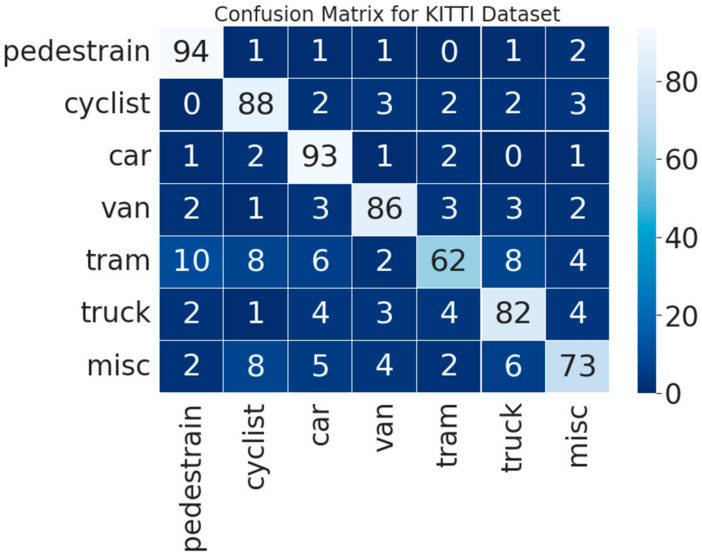
Confusion matrix of 7 classes on the KITTI database via ANN.

**Figure 12:**
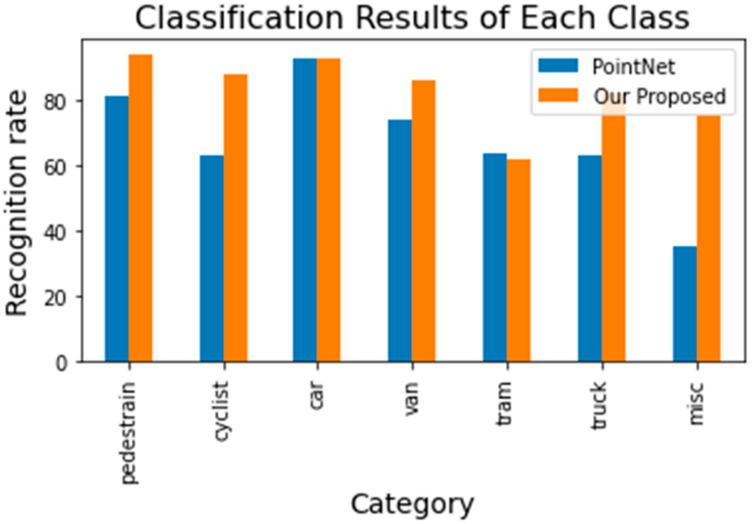
Comparison of our proposed MTMS with PointNet [39].

The confusion matrix of the KITTI-7 dataset achieved a mean recognition rate of 82.85%, presented in Figure 11. Figure 12 shows the classification result of each category of the proposed MTMS with PointNet.

The following are the limitations of the proposed MSTM system.

- The drawback of the proposed method is that for each camera data feed, a substantial amount of parameter adjusting is needed to achieve optimal performance.
- Second, it is assumed that the automobiles in the scenes need to be visible and not hidden.
- Another shortcoming is its inability to distinguish extremely small automobiles, which requires the use of multimodal information to track objects.

## 5. Conclusions

This research proposes an improved method to detect and recognize automobiles in the MTMS system. The following are the main significances of the proposed MSTM:

- Different preprocessing techniques, such as frame conversion and background subtraction, are utilized. The detection and recognition mof moving automobiles employ denoising and background subtraction techniques in which the background is modeled based on the behavioral study of intensity transitions.
- Second, we extracted the ROI to detect the vehicle’s silhouette. The recognition of automobiles is processed through an area of interest determined by the objects’ shape.
- After that, feature extraction was conducted, and two important features were abstracted. These features include energy and dense optical flow features.
- Next, the graph-mining optimizing strategy was developed to reduce the redundant features and improve the system’s performance.
- Finally, essential features are served to artificial neural networks (ANN) to detect and recognize automobiles in a robust manner.
- The proposed method was evaluated using two benchmark datasets. The proposed MSTM method yields better results with adequate speed for less restrictive highway video surveillance than other state-of-the-art systems. Moreover, enhancements should be made to tackle the occlusion issues in the scenarios.
- In future research, we will analyze image enhancement strategies and feature abstraction from low-quality image sequences.

## Figures and Tables

**Table 1:** Experimental results on LISA LDB1 dataset.

Recognition/Detection System	TPR	FDR	TP/Sequence	FP/Sequence	FP/Object
Passively trained [38]	89.5%	51.1%	4.2	4.1	0.9
Active trained [38]	93.5%	7.1%	0.3	4.2	0.1
ALVeRT [38]	95.0%	6.4%	0.29	4.2	0.06
Proposed MTMS	95.62%	4.2%	4.2	0.25	0.02

**Table 2:** Experimental results on LISA LDB2 dataset.

Recognition/Detection System	TPR	FDR	TP/Sequence	FP/Sequence	FP/Object
Passively trained [38]	83.5%	79.7%	4.0	1.0	3.3
Active trained [38]	80.5%	41.7%	0.72	0.98	0.57
ALVeRT [38]	91.7%	25.5%	0.39	1.14	0.31
Proposed MTMS	91.89%	13.14%	1.25	0.22	0.26

**Table 3:** Comparison of the evaluation metrics (precision, recall, and F1 score) of the MTSM model from the KITTI dataset.

Methods	ANN		
Activities	Precision	Recall	F1 Score
**K1**	0.846	0.940	0.891
**K2**	0.807	0.880	0.842
**K3**	0.815	0.930	0.869
**K4**	0.860	0.860	0.86
**K5**	0.826	0.620	0.708
**K6**	0.803	0.820	0.811
**K7**	0.820	0.730	0.772
**Mean**	0.825	0.826	0.821

K1 = pedestrian; K2 = cyclist; K3 = walking downstairs; K4 = sitting; K5 = standing; K6 = laying, K7 = misc.

## Data Availability

Not applicable.

## References

[1] Lincy R.B., Gayathri R. (2021). Optimally configured convolutional neural network for Tamil Handwritten Character Recognition by improved lion optimization model. Multimed. Tools Appl..

[2] Shaffi N., Hajamohideen F. (2021). uTHCD: A new benchmarking for Tamil handwritten OCR. IEEE Access.

[3] Jency R.J., Babitha L.R., Al-Heety A.T. (2021). Moving vehicle detection from video sequences for traffic surveillance system. ITEGAM-JETIA.

[4] Abdusalomov A., Mukhiddinov M., Djuraev O., Khamdamov U., Whangbo T.K. (2020). Automatic Salient Object Extraction Based on Locally Adaptive Thresholding to Generate Tactile Graphics. Appl. Sci..

[5] Al Gharrawi H., Yaghoub M.B. (2022). Traffic Management in Smart Cities Using the Weighted Least Squares Method. arXiv.

[6] Ur Rehman M.A., Raza H., Akhter I. Security Enhancement of Hill Cipher by Using Non-Square Matrix Approach. Proceedings of the 4th International Conference on Knowledge and Innovation in Engineering, Science and Technology.

[7] Kaushek K.T.R., Thiruvikkraman S., Gokul R., Nirmal A., Karthika R. Evaluating the scalability of a multi-object detector trained with multiple datasets. Proceedings of the 2021 5th International Conference on Intelligent Computing and Control Systems (ICICCS).

[8] Bhargavi D., Coyotl E.P., Gholami S. (2022). Knock, knock. Who’s there?—Identifying football player jersey numbers with synthetic data. arXiv.

[9] Wang Q., Xu N., Huang B., Wang G. (2022). Part-Aware Refinement Network for Occlusion Vehicle Detection. Electronics.

[10] Gholami S., Khashe S. (2022). Alexa, Predict My Flight Delay. arXiv.

[11] Hamdy A., Ezzat G. (2022). Deep mining of open source software bug repositories. Int. J. Comput. Appl..

[12] Maity S., Bhattacharyya A., Singh P.K., Kumar M., Sarkar R. (2022). Last Decade in Vehicle Detection and Classification: A Comprehensive Survey. Arch. Comput. Methods Eng..

[13] Chen Q., Wang W., Wu F., De S., Wang R., Zhang B., Huang X. (2019). A survey on an emerging area: Deep learning for smart city data. IEEE Trans. Emerg. Top. Comput. Intell..

[14] Di X., Shi R. (2021). A survey on autonomous vehicle control in the era of mixed-autonomy: From physics-based to AI-guided driving policy learning. Transp. Res. C Emerg. Technol..

[15] Clady X., Negri P., Milgram M., Poulenard R. Multi-class vehicle type recognition system. Proceedings of the IAPR Workshop on Artificial Neural Networks in Pattern Recognition.

[16] Petrovic V.S., Cootes T.F. Analysis of Features for Rigid Structure Vehicle Type Recognition. Proceedings of the BMVC.

[17] Negri P., Clady X., Milgram M., Poulenard R. An oriented-contour point based voting algorithm for vehicle type classification. Proceedings of the 18th International Conference on Pattern Recognition (ICPR’06).

[18] Kazemi F.M., Samadi S., Poorreza H.R., Akbarzadeh-T M.-R. Vehicle recognition based on fourier, wavelet and curvelet transforms—A comparative study. Proceedings of the Fourth International Conference on Information Technology (ITNG’07).

[19] Zhang B. (2012). Reliable classification of vehicle types based on cascade classifier ensembles. IEEE Trans. Intell. Transp. Syst..

[20] Hannan M.A., Gee C.T., Javadi M.S. (2015). Automatic vehicle classification using fast neural network and classical neural network for traffic monitoring. Turk. J. Electr. Eng. Comput. Sci..

[21] Satzoda R.K., Trivedi M.M. (2016). Looking at vehicles in the night: Detection and dynamics of rear lights. IEEE Trans. Intell. Transp. Syst..

[22] Qian Z., Yang J., Duan L. (2013). Multiclass vehicle tracking based on local feature. Proceedings of 2013 Chinese Intelligent Automation Conference.

[23] Shujuan S., Zhize X., Xingang W., Guan H., Wenqi W., De X. Real-time vehicle detection using Haar-SURF mixed features and gentle AdaBoost classifier. Proceedings of the 27th Chinese Control and Decision Conference (2015 CCDC).

[24] Elkerdawi S.M., Sayed R., ElHelw M. (2014). Real-time vehicle detection and tracking using Haar-like features and compressive tracking. ROBOT2013: First Iberian Robotics Conference.

[25] Miller N., Thomas M.A., Eichel J.A., Mishra A. A hidden Markov model for vehicle detection and counting. Proceedings of the 2015 12th Conference on Computer and Robot Vision.

[26] Sun D., Watada J. Detecting pedestrians and vehicles in traffic scene based on boosted HOG features and SVM. Proceedings of the 2015 IEEE 9th International Symposium on Intelligent Signal Processing (WISP).

[27] Laopracha N., Sunat K., Chiewchanwattana S. (2019). A novel feature selection in vehicle detection through the selection of dominant patterns of histograms of oriented gradients (DPHOG). IEEE Access.

[28] Girshick R., Donahue J., Darrell T., Mali J.K. Rich feature hierarchies for accurate object detection and semantic segmentation. Proceedings of the IEEE Conference on Computer Vision and Pattern Recognition.

[29] Sermanet P., Eigen D., Zhang X., Mathieu M., Fergus R., LeCun Y. (2013). Overfeat: Integrated recognition, localization and detection using convolutional networks. arXiv.

[30] Yu Y., Yu M., Yan G., Zhai Y. (2011). Length-based vehicle classification in multi-lane traffic flow. Trans. Tianjin Univ..

[31] Meher S.K., Murty M.N. (2013). Efficient method of moving shadow detection and vehicle classification. AEU-Int. J. Electron. Commun..

[32] Moutakki Z., Ouloul I.M., Afdel K., Amghar A. (2017). Real-time video surveillance system for traffic management with background subtraction using codebook model and occlusion handling. Transp. Telecommun..

[33] Velazquez-Pupo R., Sierra-Romero A., Torres-Roman D., Shkvarko Y.V., Santiago-Paz J., Gómez-Gutiérrez D., Robles-Valdez D., Hermosillo-Reynoso F., Romero-Delgado M. (2018). Vehicle Detection with Occlusion Handling, Tracking, and OC-SVM Classification: A High Performance Vision-Based System. Sensors.

[34] Chakrabarti D., Faloutsos C. (2006). Graph mining: Laws, generators, and algorithms. ACM Comput. Surv..

[35] Zerroug E., Belaidi S., Chtita S. (2021). Artificial neural network-based quantitative structure–activity relationships model and molecular docking for virtual screening of novel potent acetylcholinesterase inhibitors. J. Chin. Chem. Soc..

[36] Jensen M.-B., Philipsen M.-P., Møgelmose A., Moeslund T.-B., Trivedi M.-M. (2016). Vision for Looking at Traffic Lights: Issues, Survey, and Perspectives. IEEE Trans. Intell. Transp. Syst..

[37] Garcia-Garcia A., Orts-Escolano S., Oprea S., Villena-Martinez V., Garcia-Rodriguez J. (2017). A review on deep learning techniques applied to semantic segmentation. arXiv.

[38] Sivaraman S., Trivedi M.M. (2010). A general active-learning framework for on-road vehicle recognition and tracking. IEEE Trans. Intell. Transp. Syst..

[39] Qi C.R., Su H., Mo K., Guibas L.J. Pointnet: Deep Learning on Point Sets for 3d Classification and Segmentation. Proceedings of the IEEE Conference on Computer Vision and Pattern Recognition.

